# Awareness, Concerns, and Protection Strategies Against Blood-Borne Viruses Among Emergency Medicine Staff

**DOI:** 10.7759/cureus.31479

**Published:** 2022-11-14

**Authors:** Amirah F Aloushan, Falwah Alharthi, Mohammed I Alismail, Abdulrahman A Alhaqbani, Ibrahim H Almutairi, Shahad Alashgar, Noura Ahmed, Ftoon Alebrahaimi

**Affiliations:** 1 Emergency Medicine, King Abdulaziz Medical City Riyadh, Riyadh, SAU; 2 Emergency Medicine, King Saud Bin Abdulaziz University, Riyadh, SAU

**Keywords:** saudi arabia, emergency department, blood borne viruses, concerns, awareness

## Abstract

Objectives

The present study aimed to evaluate the awareness and measures taken to prevent infections of blood-borne pathogens (BBPs) among emergency department (ED) staff at King Abdulaziz Medical City in Riyadh.

Method

A cross-sectional research approach was adopted. The study recruited a sample of 200 ED medical staff from King Abdul Aziz Medical City in Riyadh city. A self-filled questionnaire was used to elicit data related to awareness and concerns, in addition to the protection strategies adopted by the ED medical staff. The gathered data was analyzed using the Statistical Package of Social Sciences (SPSS, v. 26).

Results

The study found that 42.5% (n=85) of the emergency room (ER) staff did not use regular eyewear at all, 30% (n=60) did not use face shields at all, and about 75.5% of the enrolled ED staff successfully converted to double-gloving. In addition, it was found that patients with active AIDS (64%), patients with active hepatitis (60.5%), and patients with known HIV infection (60%) were the most reported factors influencing the decision to use double-gloving. Moreover, it was found that the highest reported reasons for not double-gloving were that double-gloving is not necessary (56%, n=112) and double-gloving decreases hand sensation (31%, n=62).Finally, the study found that the most reported reasons for not using barriers other than gloves precautions were non-necessity of barriers other than gloves (31% (n=62) and non-availability (26.5%, n=53).

Conclusion

The study concluded that ER medical staff in King Abdul-Aziz Medical City in Riyadh perceived a high level of lifetime risk of infection when performing procedures and that there is a lack of education and awareness of ER staff related to using personal protective equipment (PPE) and double-gloving when performing procedures in the ER.

## Introduction

Blood-borne pathogens (BBPs) are infectious microorganisms in human blood that can transmit to other humans and cause different diseases. The main concerns of these pathogens are human immunodeficiency virus (HIV), hepatitis B virus (HBV), and hepatitis C virus (HCV), but it is not limited to these microorganisms as many pathogens are infectious through blood contact [1]. According to World Health Organization (WHO), the risk of infection from BBPs is proportional to the increase in disease prevalence in the population and the nature and frequency of exposures. In the year 2000, WHO estimated that about three million healthcare workers had percutaneous injuries worldwide, which caused 15,000 HCV, 70,000 HBV, and 500 HIV infections, with more than 90% of these infections occurring in developing countries. Infections can result from percutaneous injury (needlestick or another sharp injury, mucocutaneous injury - splash of blood or other body fluids into the eyes, nose, or mouth), or blood contact with non-intact skin. The most common and most likely to result in infection is needlestick injury. At the same time, the most common causes of needlestick injury are two-handed recapping and the unsafe collection and disposal of sharp waste [2]. Moreover, a mucocutaneous injury could take the form of contacting blood splashes or exposure to other body fluids. The exposure could be into the eyes, nose, or mouth among emergency department staff, especially in the settings of trauma or blood testing. It is less common when compared to sharp injuries but cannot be neglected [2]. In a South Korean study, mucocutaneous exposures were 13.2% of all blood exposures, and 13.3% were in the emergency department [3]. Moreover, a study conducted in multiple tertiary hospitals in Ethiopia found that blood splash (67.6%) was the main cause of blood exposure [4]. A cross-sectional survey done in two hospitals in Beijing on healthcare workers found that 48.7% had experienced exposure by contact with non-intact skin during their work lifetime. Based on their findings, on average, they had almost two episodes in 2013 alone [5].

Emergency department staff often face acute traumatic cases where contact with blood and other bodily fluids is more often seen. Locally, a study conducted by the Ministry of Health Hospitals of Saudi Arabia estimated that the emergency room of Saudi hospitals had a significantly higher rate of percutaneous injuries (18.2%) compared to US hospital emergency departments (8.7%) [6]. A study conducted in the Al Ahsa governorate from the year 2016 to 2018 found that the emergency department was the second-highest department in reporting sharp injuries among staff [7]. Another study that was conducted in Central Hospital Buraydah demonstrated that only 14 (19.2%) sharp injuries were reported out of 73 total injuries [8]. Despite the declining prevalence of HBV and HCV in Saudi Arabia by more than 50% in the last two decades, they are still a major health concern because of their potential life-threatening effects [9]. Furthermore, 10,217 new HIV cases were reported in Saudi Arabia between the years 2000 and 2009, with an average annual incidence of less than four per 100,000 [10].

Therefore, considering all means of blood exposure, including sharp object injury and mucocutaneous exposures, should give us a better understanding of ER staff exposure to blood-borne pathogens and how to prevent such incidents. Our research aims to evaluate knowledge and measures taken to prevent infections of BBPs among ED staff.

## Materials and methods

This study was conducted in the Emergency Department at King Abdulaziz Medical City in Riyadh (KAMC-R), Saudi Arabia. The study was approved by the Institutional Review Board (IRB) at King Abdullah International Medical Center (IRB Approval no. IRB/1132/22). A questionnaire was sent via email and messages and personal distribution by the researchers to emergency consultants, residents, staff, and nurses.

Study subjects

The study subjects included all emergency consultants, residents, staff, and nurses working at KAMC-R from October 2021 to March 2022. However, ER consultants, residents, staff, and nurses who were no longer working in KAMC-R from October 2021 to March 2022 and pediatric consultants, residents, staff, nurses, and other healthcare workers in the emergency department (patient care technicians, admin, medical students, interns) were excluded.

Study design

This was a cross-sectional study with a quantitative structured questionnaire. The questionnaire was sent via email and messages and personal distribution to all emergency consultants, residents, staff, and nurses at KAMC-R working from October 2021 to March 2022 to evaluate knowledge and measures taken to prevent infections of BBPs among ED staff.

Sample size

The sample size was calculated using Raosoft online calculator. Assuming a 5% margin of error and a 95% confidence level, the population sample size was calculated to be 50% of responses (the number of emergency staff at KAMC-R is 385). The minimum required sample size was calculated to be 193. However, a total of 200 participants were enrolled in this study.

Data collection tools

The questionnaire was sent by email and messages to ER consultants, residents, nurses, and staff working in KAMC-R. Due to an inadequate response, personal interviews were then conducted. The questionnaire was sent as an electronic Google form, as well as a hard copy, due to a lack of responses. The questionnaire was adapted and modified from two previous studies [11,12], where permission was obtained from the original authors. The questionnaire included demographic information, such as age, gender, subspecialty, and years of experience. It also addressed the risk of transmission, awareness of double-gloving, and reporting patterns of needlestick injuries and reasons for not double-gloving.

Data analysis

To analyze data, we used the Statistical Package of Social Sciences (SPSS, v.26.0, IBM Inc., Armonk, USA). Descriptive statistics such as frequencies, percentages, means, and standard deviations were used to analyze the participants' sociodemographic characteristics, their beliefs, attitudes, and willingness to practices that are related to using preventive strategies. The data were presented either as tables or texts. A significance level of ≤0.05 was used as the statistical significance threshold in this study.

## Results

A total of 200 ED medical staff were recruited in this study. The results presented in Table 1 represent the sociodemographic characteristics of the enrolled ED staff. The results showed that the mean age of the enrolled participants was 33.5±7.68 and the mean years of practice were 8.5±6.98. It was found that nurses represented the highest category (52.5%, n=105), followed by residents (30%, n=60). In addition, 10.5% (n=21) were staff, 4% (n=8) were assistant consultants, 20.5% (n=5) were consultants, and 0.5% (n=1) were associate consultants.

**Table 1:** Sociodemographic characteristics of the enrolled emergency room staff (n=200)

Variable	Female	Male	Total
Mean age (years) ± standard deviation (SD)	34.8±7.95	31.9±7.04	33.5±7.68
Mean years of practice ± SD	10.0±7.05	6.7±6.49	8.5±6.98
Position			
Consultant	1 (0.9)	4 (4.4)	5 (2.5)
Associate consultant	1 (0.9)	0 (0)	1 (0.5)
Assistant consultant	3 (2.7)	5 (5.6)	8 (4)
Staff	4 (3.6)	17 (18.9)	21 (10.5)
Resident	16 (14.5)	44 (48.9)	60 (30)
Nurse	85 (77.3)	20 (22.2)	105 (52.5)

The results presented in Table 2 show the proportion of procedures for which the emergency room (ER) staff reported using barrier precautions. The results showed that 42.5% (n=85) of the ER staff did not use regular eyewear at all, 22% (n=44) used them in 25% of their procedures, 15.5% (n=31) used them in 50% of their procedures, 8.5% (n=17) used them in 75% of their procedures, and 11.5% (n=23) used them in 100% of their procedures.

**Table 2:** Proportion of procedures for which emergency department staff reported using barrier precautions (n=200)

Barrier precaution/percentage	0%	25%	50%	75%	100%
Regular eyewear	85 (42.5%)	44 (22%)	31 (15.5%)	17 (8.5%)	23 (11.5%)
Face shields attached to surgical masks	60 (30%)	70 (35%)	32 (16%)	14 (7%)	24 (12%)
Goggles	93 (46.5%)	61 (30.5%)	29 (14.5%)	10 (5%)	7 (3.5%)
Full face shields	79 (39.5%)	59 (29.5%)	34 (17%)	14 (7%)	14 (7%)
Double- or triple-gloving	47 (23.5%)	45 (22.5%)	45 (22.5%)	39 (19.5%)	24 (12%)

As shown in Table 2, with regard to the face shields attached to surgical masks, it was found that 30% (n=60) did not use them at all, 35% (n=70) used them in 25% of their procedures, 16% (n=32) used them in 50% of their procedures, 7% (n=14) used them in 75% of their procedures, and 12% (n=24) used them in 100% of their procedures.

For goggles, it was found that 46.5% (n=93) did not use them at all in their procedures, 30.5% (n=61) used them in 25% of their procedures, 14.5% (n=29) used them in 50% of their procedures, 5% (n=10) used them in 75% of their procedures, and 3.5% (n=7) used them in 100% of their procedures as shown in Table 2.

The results related to using the full face shield it was found that 39.5% (n=79) did not use them at all in their procedures, 29.5% (n=59) used them in 25% of the procedures, 17% (n=34) used them in 50% of the procedures, 7% (n=14) used them in 75% of the procedures, and 7% (n=14) used them in all procedures they performed as presented in Table 2.

Finally, the results in Table 2 showed that 23.5% (47) did not use double- or triple-gloving at all in the procedures they performed, 22.5% (n=45) used them in 25% of the procedures, 22.5% (n=45) used them in 50% of the procedures, 19.5% (n=39) and 12% (n=24) used them in 75% and 100% of the procedures they performed, respectively.

The results presented in Table 3 show the ED staff practices related to double-gloving. The results showed that 75.5% (n=151) converted to double-gloving successfully, whereas 24.5% (n=49) were not successful in conversion to double-gloving. It was found that the mean time in days to convert to double-gloving among those who were successful (n=151) was 51.2±296.9. Moreover, the results showed that 54.3% (n=82) believed that using double gloves decreases hand sensation.

**Table 3:** The emergency room staff's practices and beliefs related to double-gloving (n=200)

Variable	n (%)
Conversion to double gloves	
Successful	151 (75.5%)
Non-successful	49 (24.5%)
Mean time to successful conversion to double gloves	51.2±296.9
Do you believe that using double gloves decreases your hand sensation?	
Yes	82 (54.3)
No	69 (45.7)

The results presented in Table 4 show that ED staff responses are related to the factors influencing the decision to double-gloving. The results revealed that the patient with active AIDS was ranked first (3.29±1.18), followed by the patient with known HIV Infection (3.22±1.20) in the second rank, and the last two ranked factors were patient's gender (1.00±1.35) and patient's marital status (0.95±1.26).

**Table 4:** The emergency department staff responses related to the factors influencing the decision to double-gloving

Factor	Extremely important	Very important	Moderately important	Slightly important	Not important	Mean ± SD	Rank
Patient's gender	13 (6.5)	25 (12.5)	29 (14.5)	15 (7.5)	118 (59)	1.00±1.35	11
Patient's race	16 (8)	22 (11)	32 (16)	13 (6.5)	117 (58.5)	1.03±1.38	10
Patient's age	15 (7.5)	32 (16)	28 (14)	21 (10.5)	104 (52)	1.17±1.40	9
Patient's marital status	9 (4.5)	22 (11)	34 (17)	19 (9.5)	116 (58)	0.95±1.26	12
Hospital	38 (19)	39 (19.5)	35 (17.5)	24 (12)	64 (32)	1.82±1.53	8
Type of surgery	55 (27.5)	61 (30.5)	35 (17.5)	13 (6.5)	36 (18)	2.43±1.42	7
Trauma case	62 (31)	54 (27)	38 (19)	16 (8)	30 (15)	2.51±1.40	6
Patients with known IV drug user	90 (45)	47 (23.5)	32 (16)	7 (3.5)	24 (12)	2.86±1.35	5
Patients with known HIV infection	120 (60)	40 (20)	20 (10)	5 (2.5)	15 (7.5)	3.22±1.20	2
Patients with known hepatitis carrier	113 (56.5)	40 (20)	20 (10)	13 (6.5)	14 (7)	3.13±1.24	4
Patients with active AIDS	128 (64)	36 (18)	16 (8)	6 (3)	14 (7)	3.29±1.18	1
Patients with active hepatitis	121 (60.5)	37 (18.5)	18 (9)	9 (4.5)	15 (7.5)	3.20±1.23	3

The results presented in Table 5 represent the ED staff's responses related to sustaining and reporting needlestick injuries. The results showed that 30% (n=60) never had any micro-abrasions or open sores/cracks on their hands or nailed region. In addition, it was found that 75.5% (n=151) never sustained a needlestick injury.

**Table 5:** The emergency department staff responses related to sustaining and reporting needlestick injuries

Item	n (%)
Do you have any micro-abrasions or open sores/cracks on your hands or nailed region?	
Always	8 (4)
Sometimes	33 (16.5)
Occasionally	27 (13.5)
Rarely	72 (36)
Never	60 (30)
How often do you sustain a needlestick injury?	
None	151 (75.5)
Once a week	11 (5.5)
Once a month	2 (1)
Once a year	36 (18)
How often do you report an actual needlestick injury?	
Always	68 (34)
Sometimes	6 (3)
Occasionally	7 (3.5)
Rarely	34 (17)
Never	85 (42.5)
How many needlestick injuries have you had in the last three years?	
0 - 3	197 (98.5)
More than 3	3 (1.5)
How often do you sustain a skin break from other means in the emergency room?	
Always	13 (6.5)
Sometimes	20 (10)
Occasionally	24 (12)
Rarely	76 (38)
Never	67 (33.5)
Have you ever been stuck by a needle while treating a patient positive for:	
HIV	3 (1.5)
AIDS	2 (1)
Hepatitis B	7 (3.5)
Hepatitis C	8 (4)
Mean times stuck by a needle while treating a patient positive for:	
HIV	1.98±0.12
AIDS	1.99±0.10
Hepatitis B	1.97±0.18
Hepatitis C	1.96±0.20
Secondary to a needlestick injury, have you ever been treated with	
Azidothymidine (AZT, zidovudine)	3 (1.5)
Gammaglobulin	3 (1.5)

With regard to reporting needlestick injury, it was found that 34% (n=68) always report, 3% (n=6) sometimes report, 3.5% (n=7) occasionally report, 17% (n=34) rarely report, and 42.5% (n=85) never reported an actual needlestick injury. The results in Table 5 showed that 98.5% (n=197) had none to three needlestick injuries in the last three years, whereas only 1.5% (n=3) had more than three needlestick injuries in the last three years.

The results shown in Table 5 revealed that 33.5% (n=67) never sustained a skin break from other means in the emergency room, whereas 38% (n=76), 12% (n=24), 12% (n=24), and 6.5% (n=13) rarely, occasionally, sometimes, and always sustained a skin break from other means in the emergency room, respectively.

The results in Table 5 revealed that 1.5% (n=3) had ever been stuck by a needle while treating a patient positive for HIV, 1% (n=2) had ever been stuck by a needle while treating a patient positive for AIDS, 3.5% (n=7) had ever been stuck by a needle while treating a patient positive for hepatitis B, and 4% (n=8) had ever been stuck by a needle while treating a patient positive for hepatitis C. In addition, it was found that the mean times stuck by a needle while treating a patient positive for HIV was 1.98±0.12, for AIDS was 1.99±0.10, for hepatitis B was 1.97±0.18, and for hepatitis C was 1.96±0.20. Finally, it was found that 1.5% (n=3) of the ED staff were treated with azidothymidine (AZT, zidovudine), and 1.5% (n=3) were treated with gammaglobulin, secondary to a needlestick injury.

The results shown in Table 6 represent the reasons for not double-gloving as perceived by the ED staff. The results showed that the highest reported reasons were that double-gloving is not necessary (56%, n=112), followed by that double-gloving causes decreased hand sensation (31%, n=62), reduced ability to manipulate tissues and instruments (21%, n=42), other reasons (11%, n=22), that double-gloving made the surgery more difficult (9.5%, n=19), produced hand tingling and/or numbness (7.5%, n=15) and the lowest reported reason was that double-gloving produced hand pain (6.5%, n=13).

**Table 6:** Reasons for not double-gloving as perceived by the emergency department staff

Reason	n (%)	Rank
Not necessary	112 (56)	1
Produced hand tingling and/or numbness	15 (7.5)	6
Produced hand pain	13 (6.5)	7
Decreased hand sensation	62 (31)	2
Made surgery more difficult	19 (9.5)	5
Decreased ability to manipulate tissues and instruments	42 (21)	3
Other	22 (11)	4

The results presented in Table 7 show the reasons for not using barriers other than gloves precautions as perceived by the ED staff. The results showed that non-necessity of barriers other than gloves precautions was the highest reported reason (31% (n=62), followed by non-availability (26.5%, n=53) ranked as second, non-comfortable of these barriers (23.5%, n=47) ranked as third reason, fogging (for protective eyewear) was ranked fifth and reported by 14.5% (n=29) and interference with surgery was the lowest reported reason (4.5%, n=9).

**Table 7:** Reasons for not using barriers other than gloves precautions as perceived by the ED staff

Reason	n (%)	Rank
Not available	53 (26.5)	2
Not necessary	62 (31)	1
Not comfortable	47 (23.5)	3
Fogging (for protective eyewear)	29 (14.5)	4
Interfered with surgery	9 (4.5)	5

The results presented in Table 8 represent the emergency medicine staff's attitudes toward preventive strategies. The results showed that 47.5% (n=95) believed that patients should be routinely screened for HIV antibodies, whereas 52.5% (n=105) negatively responded to that item. In addition, it was found that 9.5% (n=19) believed that research to reduce the risk of transmission of blood-borne disease in the emergency room is too much, whereas 58% (n=116) and 32.5% (n=65) believed that research to reduce the risk of transmission of blood-borne disease in the emergency room is about right and too little, respectively.

**Table 8:** Emergency medicine staff's attitudes toward preventive strategies

Item	n (%)
Do you believe that patients should be routinely screened for HIV antibodies?	
Yes	95 (47.5)
No	105 (52.5)
Do you believe that research to reduce the risk of transmission of blood-borne disease in the emergency room is:	
Too much	19 (9.5)
About right	116 (58)
Too little	65 (32.5)
Do you believe that the effort of professional organizations to reduce the risk of transmission of blood-borne disease in the emergency room is:	
Too much	28 (14)
About right	126 (63)
Too little	46 (23)

Moreover, it was found that 14% (n=28) of the ED staff believed that the effort of professional organizations to reduce the risk of transmission of blood-borne disease in the emergency room is too much, whereas 63% (n=126) and 23% (n=46) believed that the effort of professional organizations to reduce the risk of transmission of blood-borne disease in the emergency room is about right and too little, respectively, as presented in Table 8.

The results presented in Table 9 represent the emergency medicine staff's perceived risk of infection. The results showed that 25% (n=50) rated their lifetime risk of HIV infection from occupational exposure as high, whereas 31% (n=62) rated it as a medium, 30% (n=60) rated it as low, and 14% (n=28) rated their lifetime risk of HIV infection from occupational exposure as insignificant.

**Table 9:** Emergency medicine staff's perceived risk of infection

Item	n (%)
Rate your perceived lifetime risk of HIV infection from occupational exposure	
High	50 (25)
Medium	62 (31)
Low	60 (30)
Insignificant	28 (14)
The frequency with which you receive sharp injuries in the emergency room	
0%	83 (41.5)
25%	71 (35.5)
50%	29 (14.5)
75%	10 (5)
100%	7 (3.5)
What percentage of your patients do you believe are positive for HIV?	
Less than 10%	118 (59)
10% - 50%	79 (38.5)
More than 50%	3 (1.5)
What percentage of your patients do you believe are positive for hepatitis?	
Less than 10%	68 (34)
10% - 50%	117 (58.5)
More than 50%	15 (7.5)
What percentage of your patients do you order blood tests to screen for HIV?	
Less than 10%	134 (67)
10% - 50%	60 (30)
More than 50%	6 (3)
What percentage of your patients do you order blood tests to screen for hepatitis?	
Less than 10%	105 (52.5)
10% - 50%	75 (37.5)
More than 50%	20 (10)
Have you been vaccinated against hepatitis B?	
Yes	183 (91.5)
No	17 (8.5)
Has anyone you personally know ever been infected with	
HIV	9 (4.5)
Hepatitis	35 (17.5)
None	156 (78)
Has anyone you personally know ever died from	
AIDS	8 (4)
Hepatitis	14 (7)
None	178 (89)

It is obvious from the results in Table 9 that the findings related to the frequency with which the ED staff received sharp injuries in the emergency room revealed that 41.5% (n=83) had 0% frequency, 35.5% (n=71) had 25% frequency, 14.5% (n=29) had 50% frequency, 5% (n=10) had 75% frequency, and 3.5% (n=7) had 100% frequency of receiving sharp injuries in the emergency room.

The results shown in Table 9 indicated that 59% (n=118) believed that less than 10% of their patients are positive for HIV, whereas 38.5% (n=79) believed that 10% to 50% are positive for HIV, and 1.5% (n=3) believed that more than 50% of their patients are positive for HIV.

It was found that 34% (n=68) believed that less than 10% of their patients were positive for hepatitis, 58.5% (n=117) believed that 10% to 50% of the patients were positive for hepatitis, and 7.5% (n=15) believed that more than 50% of the patients were positive for hepatitis as shown in Table 9.

Concerning the need for a blood test screening, it was found that 67% (n=134) reported that they ordered blood tests to screen for HIV for less than 10%, whereas 30% (n=60) did that for 10% to 50% of their patients and 3% (n=6) did that for more than 50% of their patients. Moreover, it was found that 52.5% (n=105) reported that they ordered blood tests to screen for hepatitis for less than 10% of their patients, 37.5% (n=750 did that for 10% to 50% of their patients, and 10% (n=20) did that for more than 50% of their patients as shown in Table 9.

The results shown in Table 9 revealed that 91.5% (n=183) of the ED staff had been vaccinated for hepatitis B. In addition, it was shown that 4.5% (n=9) and 17.5% (n=35) personally knew individuals infected with HIV and hepatitis, respectively. On the other hand, 78% (n=156) negatively responded to this item. Finally, it was found that 4% (n=8) and 7% (n=14) personally knew individuals who died from AIDS and hepatitis, respectively, and 89% (n=178) never knew patients who died either from AIDS or hepatitis.

The results presented in Table 10 represent the ED staff's willingness to adopt preventive strategies. The results showed that 77.5% (n=155) pointed their willingness to change the way they perform procedures proven if preventive strategies were made available, 81.5% (n=163) were willing to hand back of suture needles with the needle tip clamped in the needle holder to prevent injuries from free sharps. Finally, it was found that 75% (n=150) were willing to return sharps in a kidney basin to prevent injuries due to instrument passage.

**Table 10:** The emergency department staff's willingness to adopt preventive strategies

Item	n (%)
Would you change the way you performed procedure-proven preventive strategies were made available?	
Yes	155 (77.5)
No	45 (22.5)
Will you use any of the following specific strategies to prevent the most frequent mechanisms of sharp injury?	
Handing back of suture needles with the needle tip clamped in the needle holder (to prevent injuries from free sharps)	
Yes	163 (81.5)
No	17 (8.5)
Maybe	20 (10)
Returning sharps in a kidney basin (to prevent injuries due to instrument passage)	
Yes	150 (75)
No	28 (14)
Maybe	22 (11)

## Discussion

The present study aimed to evaluate awareness and measures taken to prevent infections of BBPs among ED staff at King Abdul-Aziz Medical City in Riyadh. Our findings revealed that there is a significant non-adherence to using barrier precautions when performing procedures among ED staff. This result might be referred to the lack of knowledge, awareness, and negative perceptions of the ED staff related to the adherence and compliance to using barrier precautions to avoid catching any kind of infection. However, our findings suggested that there is a high rate of conversion to double-gloving among the ED staff, but this was accompanied by a relatively long time period, exceeding 51 days, to perform that, which might be referred to a lack of education or awareness sessions provided for healthcare workers regarding the double-gloving. Further, this result might be attributed to the data flow shortage related to the beneficial effects of using double-gloving when performing medical procedures.

In addition, this might be referred to the policies and procedures that require minimum use of barrier precautions, not to the extent of using double-gloving, especially since the study sample is ED staff. Moreover, the non-successful conversion to double-gloving might be explained by the fact that about half of the respondents believed that double-gloving is decreasing their hand sensation, which could be affecting the medical procedures they are performing and hence causing specific medical errors. These findings are consistent with the findings reported by AlJehani et al. [13], who found that a great majority of surgeons in King Abdul-Aziz Hospital were successful in conversion to double-gloving.

In a study by Al-Faleh [9], it was evidenced that double-gloving significantly improved the management of BBVs, especially hepatitis viral infections. Our findings showed that patients with active AIDS, patients with known HIV infection, and patients with active hepatitis were the most reported factors influencing the ED staff's decision to double-gloving. This result might be referred to the ED staff's concerns related to these types of infections and the high risk they are exposed to when dealing with a patient having either AIDS, HIV, or hepatitis in the ED. These results are consistent with the findings reported by Obalum et al. [14], who found that concerns related to the risk of infection by HIV, AIDS, or hepatitis patients were the most influencing the orthopedic surgeons' decision to double-gloving.

Furthermore, our findings showed that there is a significant lack of reporting needlestick injuries among the ED staff. This might be explained by the result that the majority of the recruited ED staff never sustained a needlestick injury. We evidenced that 98.5% of the recruited ED staff had less than three needlestick injuries within the last three years. In addition, it was found that a very low proportion of the respondents were stuck by a needlestick injury when treating patients with HIV, hepatitis B, or hepatitis C. This might be referred to that dealing with those patients was the most influential factor for double-gloving among the recruited ED staff. Moreover, the previous finding is supported by the evidence that a very low proportion of the respondents were treated with oral zidovudine (AZT) or intravenous immunoglobulin. These results are in accordance with the findings reported by Sriram [15], who indicated that a low proportion of healthcare workers in India are exposed to needlestick injuries in a teaching hospital.

The findings of this study revealed that non-necessity was the highest reported reason for not double-gloving or even using the precaution barriers other than the double-gloving among the recruited ED staff. This result might be attributed to the lack of knowledge and awareness among the ED staff about using personal protective equipment (PPEs). Our findings are not in line with the findings reported by Aljehani et al. [13], who indicated that requiring double-gloving for special cases is the most factor influencing the use of double-gloving. 

Our findings indicated that the ED staff had specific concerns related to the risk they might be exposed to while working in the ED. It was evidenced that 95% of respondents believed that patients should be routinely screened for HIV antibodies. However, they showed positive attitudes towards the amount of research and the efforts of professional organizations related to reducing the risk of transmission of blood-borne disease in the emergency room.

The results related to the ED perceived lifetime risk of infection showed that the ED staff were significantly concerned about the lifetime risk of HIV infection from occupational exposure and receiving sharps injuries in the ED. This was evidenced that 91.5% of the enrolled ED staff were vaccinated against hepatitis B and requested the patients to perform blood tests to screen for hepatitis and HIV. These findings are supported by the results reported by Shrestha et al. [16], who found that concerns about the lifetime risk of HIV infection or hepatitis were significantly observed among Nepali healthcare workers in a tertiary care hospital.

Our findings suggested that the great majority of the enrolled ED staff were willing to change the way they perform the procedure if the PPEs are made available, willing to hand back suture needles with the needle tip clamped in the needle holder, and willing to return sharps in a kidney basin, which indicates that they perceive the high risk of not using PPEs and their positive attitude towards using PPEs when performing the required procedure in the ED. These findings are supported by the results reported by Abukhelaif [17], who found that nurses working in Albaha King Fahad Hospital had positive attitudes and a remarkable willingness to use PPEs when practicing their clinical procedures.

Despite the significant findings reported in this study, there are still many constraints limiting the generalizability of the study results. One limitation is that the present study is a single-center study, which makes the findings restricted to the study setting, and thus, results cannot be generalized to other healthcare settings. Another limitation is the psychometric properties of the data collection tool. The scales used to measure the attitudes, awareness, concerns, and willingness did not have a total score that clarifies the level of these variables. Therefore, we could not numerically or categorically determine the level of attitudes, awareness, concerns, or willingness of ER medical staff.

## Conclusions

The study concluded that the ED staff in King Abdulaziz Medical City in Riyadh perceived a high level of lifetime risk of infection when performing procedures. In addition, the study concluded that there is a lack of educational and awareness support for ED staff related to using PPEs and double-gloving when performing procedures in the ED. Finally, the study recommends conducting a multi-center study that assesses the concerns, knowledge, attitudes, and willingness of the ED staff related to using personal protective equipment and barrier precautions. Also, the study recommends conducting intensive educational and training sessions about the role of PPEs in reducing and preventing the risk of infection in ED settings.
